# Bioprospecting of Less-Polar Fractions of *Ericaria crinita* and *Ericaria amentacea*: Developmental Toxicity and Antioxidant Activity

**DOI:** 10.3390/md20010057

**Published:** 2022-01-06

**Authors:** Sanja Radman, Lara Čižmek, Sanja Babić, Ana-Marija Cikoš, Rozelindra Čož-Rakovac, Stela Jokić, Igor Jerković

**Affiliations:** 1 Department of Organic Chemistry, Faculty of Chemistry and Technology, University of Split, Ruđera Boškovića 35, 21000 Split, Croatia; sanja.radman@ktf-split.hr; 2 Laboratory for Aquaculture Biotechnology, Division of Materials Chemistry, Ruđer Bošković Institute, Bijenička Cesta 54, 10000 Zagreb, Croatia; lara.cizmek@irb.hr (L.Č.); sanja.babic@irb.hr (S.B.); rozelindra.coz-rakovac@irb.hr (R.Č.-R.); 3 Department of Process Engineering, Faculty of Food Technology, Josip Juraj Strossmayer University of Osijek, Franje Kuhača 18, 31000 Osijek, Croatia; acikos@ptfos.hr (A.-M.C.); sjokic@ptfos.hr (S.J.)

**Keywords:** brown algae, UHPLC-ESI-HRMS, fatty acid amides, fucoxanthin, pheophytin *a*, *b* and their derivatives, terpenes, zebrafish embryotoxicity, antioxidant activity

## Abstract

*Ericaria crinita* and *Ericaria amentacea* from the Adriatic Sea (Croatia) were investigated with respect to the presence of less-polar compounds for the first time after fractionation by solid-phase extraction (SPE). The composition of less-polar fractions of freeze-dried *E. crinita* (FdEc) and *E. amentacea* (FdEa) were analyzed by high-performance liquid chromatography–high-resolution mass spectrometry with electrospray ionization (UHPLC-ESI-HRMS). The major identified compounds were: amides of higher aliphatic acids (palmitoleamide, linoleamide, palmitamide, oleamide and erucamide) and related compounds, carotenoid (fucoxanthin), chlorophyll derivatives (pheophytin *a* and *b* and their derivatives) and higher terpenes (loliolide, isoamijiol with its oxidation product), β-stigmasterol and (3β,6α)-14-methylergosta-8,24(28)-diene-3,6-diol). The toxic effects observed on the less-polar fractions obtained from *Ericaria* species on zebrafish *Danio rerio* embryos could be associated with the high abundance of all five detected amides. The antioxidant activity of the fractions was evaluated by means of five independent assays, including the reduction of the radical cation (ABTS), the oxygen radical absorbance capacity (ORAC), ferric-reducing antioxidant power (FRAP), the 2,2-diphenyl-1-picryl-hydrazyl (DPPH) assay and the Folin–Ciocalteu method. A higher antioxidant activity of *E. amentacea* in comparison to that of the *E. crinita* fractions was found with IC_50_ concentrations of 0.072 and 1.177 mg/mL, respectively. The correlation between the activity and the chemical composition revealed that the synergistic effect of different compounds impacted their antioxidant response.

## 1. Introduction

*Cystoseira* species are among the most important foundation species in the Mediterranean, forming complex habitats essential for biodiversity and ecosystem functioning [1,2]. Most of these species have been protected (Annex II of the Barcelona Convention, COM/2009/0585 FIN [3], Annex I of the Bern Convention [4] and the Habitat Directive [5]). In the Mediterranean Sea, about two-thirds of around 28 *Cystoseira* species are considered to be endemic [6].

Orellana et al. [7] concluded that the genus *Cystoseira* was polyphyletic and proposed the reinstatement of the genera for the species of the genus outside the clade of *Cystoseira sensu stricto* in order to achieve monophyly in the group classification. Molinari-Novoa et al. [8] reinvestigated the genera *Ericaria* Stackhouse and determined that *Ericaria* are the correct names for the “Cystoseira 2” and “Cystoseira 3” clades, as previously defined by Orellana et al. [7]. Consequently, *Ericaria crinita* (Duby) Molinari & Guiry 2020 is now accepted taxonomically instead of *Cystoseira crinita* (Duby) 1830, and *Ericaria amentacea* (C.Agardh) Molinari & Guiry 2020 is accepted as *Cystoseira amentacea* (C.Agardh) Bory.

Sterols and volatiles were investigated in *E. crinita* from the Black Sea [9]. Seven sterols were found with fucosterol as the main sterol, followed by 24-ethyl-cholest-5-en-3β-ol. The volatiles from the lipophilic extract were analyzed, and the major compounds were monoterpenes, from which dihydroactinidiolide was identified. The fatty acids (FAs) profile was investigated in *E. crinita* from Bulgarian Black Sea, and palmitic acid was found as the major compound, followed by linoleic acid and eicosapentaenoic acid [10]. The chemical composition of *E. crinita* from the Eastern Mediterranean [10] was also studied. Fourteen sterols have been identified with fucosterol as the main sterol, followed by 24-cholesterol and methyl-cholesta-5,24(28)-dien-3β-ol, while 24-ethyl-cholest-5-en-3β-ol was present only in traces and, among others, new sterol 24-nor-chol-5,22-dien-3β-ol was identified. The main FA was palmitic acid, followed by myristic and oleic acids. In the Mediterranean, the identified *E. crinita* [11] volatiles were dihydroactinidiolide, hexahydrofarnesyl acetone and 3,7-dimethylocta-1,6-diene-3-ol-2-aminobenzoate, along with the chlorinated compounds (ethanes substituted with chlorine and ethoxy groups). Nuclear magnetic resonance (NMR), mass spectrometry (MS), ultraviolet (UV) and infrared (IR) spectroscopy determined the meroterpenoids in *E. crinita* from the south coast of Sardinia [12]. Six new tetraprenyltoluquinol derivatives, two new triprenyltoluquinol derivatives, two new tetraprenyltoluquinone derivatives and four known tetraprenyltoluquinol derivatives were isolated and identified. The hydroquinones were found to have a powerful antioxidant activity, but none of the tested compounds showed antibacterial activity, while a moderate antitumor activity was found for several tested compounds. The phytochemical analysis of *E. crinita* collected from the French Riviera coast revealed the presence of new tetraprenyltoluquinols [13]. Diterpenes and sesquiterpenes from the *Cystoseria* genus were reviewed, including the data for *E. crinita* [14].

*E. amentacea* collected from the Sicilian coast has been studied [15,16,17,18,19]. Many tetraprenyltoluquinols have been isolated, either with a regular or irregular diterpenoid moiety. The regular tetraprenyltoluquinols that were found were: strictaketal, isocystoketal, isostrictaketal, isobalearone, (*Z*,*E*)-bifurcarenone, amentaepoxide and amentadione. The identified irregular or rearranged tetraprenyltoluquinols were: neobalearone and 2-epi-neobalearone. Two new cystoketal derivatives, demethoxy cystoketal chromane and cystoketal quinone, were isolated from *E. amentacea* collected from the French Riviera in addition to the sterols and previously described meroditerpenes [15]. Two new compounds were found: 4′-methoxy-(2*E*)-bifurcarenone and its chromene derivative. In a biological screening it was demonstrated that 4′-methoxy-(2*E*)-bifurcarenone inhibits cell division, since it stops the development of the fertilized eggs of the common sea-urchin *Paracentrotus lividus* [20]. DMSO and 50%-ethanolic extracts from *E. amentacea* from the Ligurian Sea (northwestern Mediterranean) were obtained, and their bioactive properties were investigated [21]. Meroditerpenes were found in both extracts with the structures containing the chromane or quinone groups, such as: cystoketal quinone, demethylcystoketal chromane and cystoketal chromane, and/or cystoketal. The extracts showed a very low grade of toxicity on RAW 264.7 macrophages and L929 fibroblasts and a plethora of antioxidant and anti-inflammatory effects [21]. The different efficacy of the two extracts in the various antioxidant and anti-inflammatory tests may be attributable to the quantitative, and not qualitative, difference of the present natural organic compounds. The FAs profile of *E. amentacea* lipid extract was characterized by high amounts of saturated FAs (SFAs), around 40.51% of the total FAs, and palmitic acid was the predominant one [22].

Although it is known that these two species contain a wide variety of secondary metabolites (terpenes and meroterpenes are described as the predominant constituents [15]), detailed compositions of their less-polar fractions have not been reported yet, and there are no reports of *E. crinita* and *E. amentacea* from the Adriatic Sea. We expect to identify pigments and other less-polar constituents after the fractionation by solid-phase extraction (SPE) and to evaluate their embryotoxic and antioxidant potential. Therefore, the main goals of the present research were to: (a) determine the composition of less-polar fractions of freeze-dried *E. crinita* (FdEc) and *E. amentacea* (FdEa) by high-performance liquid-chromatography-high-resolution mass spectrometry with electrospray ionization (UHPLC-ESI-HRMS) on two columns; (b) compare the obtained chemical profiles of FdEc and FdEa; (c) determine the fractions’ embryotoxic potential using zebrafish *Danio rerio* embryos; (d) determine the antioxidant potential of fractions obtained from *Ericaria* species by utilizing five in vitro assays (Folin-Ciocalteu, reduction of radical cation ABTS^+^, 2,2-diphenyl-1-picryl-hydrazyl (DPPH) assay, ferric-reducing antioxidant power (FRAP) and an oxygen radical absorbance capacity (ORAC) assay); and (e) discuss the observed antioxidant activity with respect to the determined composition of the fractions.

## 2. Results and Discussion

Although it is known that *E. crinita* and *E. amentacea* contain a wide variety of secondary metabolites [15], the detailed composition of their less-polar fractions has not been reported yet, and there are no reports on these algae from the Adriatic Sea. Therefore, FdEc and FdEa samples were fractionated by SPE (Section 3.3) to obtain less-polar fractions F3 and F4. The obtained fractions were analyzed by UHPLC-ESI(+)-HRMS. Two columns were used for the analysis: USP L7 (Acquity BEH C8) and USP L11 (Acquity CSH phenyl-hexyl). A slightly better separation as well as peak shapes were achieved on the Acquity CSH phenyl-hexyl column with charged surface hybrid particle technology. The major compounds (in the positive ion mode) were tentatively identified on the basis of their elemental compositions and tandem mass spectra. The major identified compounds were: amides of higher aliphatic acids, carotenoid, chlorophyll derivatives and higher terpenes.

**Figure 1:**
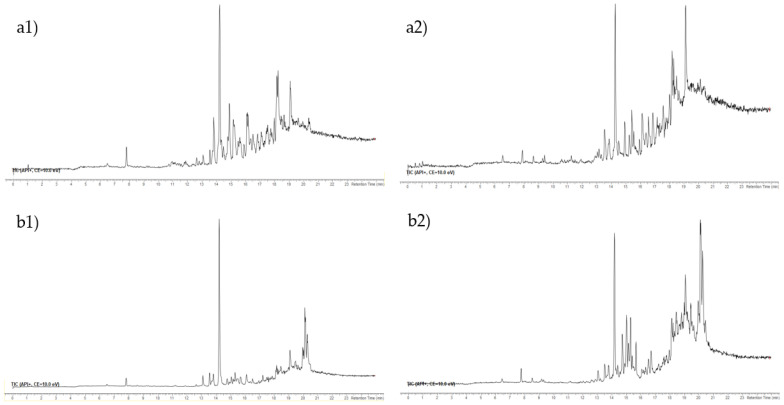
Total ion chromatogram (TIC) (**a**) F3 and (**b**) F4 of (**1**) *E. crinita* and (**2**) *E. amentacea* on Acquity CSH phenyl-hexyl column.

**Figure 2:**
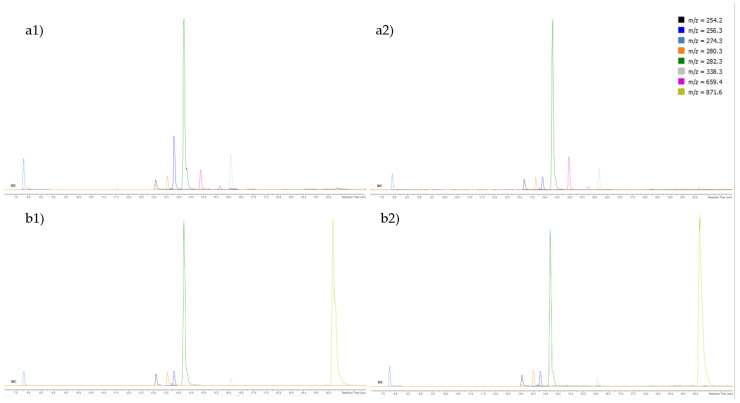
Extracted ion chromatograms (XIC) zoom of the most abundant ions in the fractions (**a**) F3 and (**b**) F4 of (**1**) *E. crinita* and (**2**) *E. amentacea* on Acquity CSH phenyl-hexyl column.

The total ion chromatograms (TICs) of the fractions F3 and F4 are shown in Figure 1, and the extracted ion chromatograms (XICs) of the most abundant ions in the fractions F3 and F4 are shown in Figure 2.

Oleamide (amide of oleic acid, entry 5, Table 1 and Table 2) was the most abundant compound in F3 of FdEc and F4 of FdEa. It has been reported as a natural compound in different natural extracts [23,24] including the algae *Rhizoclonium hieroglyphicum* [25] and *Prymnesium parvum* carter [26]. Oleamide is an endogenous bioactive signaling molecule that acts in diverse cell types and consequently triggers different biological and pharmacological effects [23,24,27,28,29]. Palmitoleamide (entry 1, Table 1 and Table 2) and palmitamide (entry 3, Table 1 and Table 2) were also present. Palmitoleamide was isolated and characterized as one of the major toxic compound exuded from cells into the surrounding environment from both laboratory-grown and field samples of *P. parvum* [26]. Palmitoleamide and octadecanamide were both identified in the essential oil from the seagrass *Zostera marina* [30]. Erucamide (entry 7, Table 1 and Table 2) was also present in both algae with a higher abundance in F3, and previously it was identified in *P. parvum* [26]. Fatty acid amides were also identified from freshwater green alga *Rhizoclonium hieroglyphicum* [25]. Another group of present fatty acid derivatives were fatty acid glycerides: 2,3-dihydroxypropyl palmitate (entry 4, Table 1 and Table 2) and 2,3-dihydroxypropyl stearate (entry 6, Table 1 and Table 2), as well as 2-hydroxypropyl stearate (entry 8, Table 1 and Table 2). Mono- and diglycerides of stearic, palmitic, oleic and arachidonic acids were found to prevail in *Fucus virsoides* FA composition [31]. In addition, 1-hydroxy-3-(tetradecanoyloxy)-2-propanyl (9*Z*)-octadec-9-enoate (entry 9, Table 1 and Table 2) and hexadecasphinganine (entry 21, Table 1 and Table 2) were found in both fractions of FdEc and FdEa. Sphinganines are dihydro derivatives of the long chain amino alcohol sphingosine that is the core moiety of sphingolipids. It was previously found as the product of *Pseudovibrio* sp. W64 marine sponge-derived bacterium [32], and it showed biological activities including antiseptic, anti-inflammatory [33] and antimicrobial activity [34].

Among xanthophyll carotenoids, only fucoxanthin (entry 18, Table 1 and Table 2) was found in F3 and F4 of both algae, being the most abundant in F3 of FdEc followed by F3 of FdEa. Remarkably lower values were found in F4 fractions of both algae. It has been reported as the main carotenoid pigment in all brown algae [35] that exhibited different biological activities, i.e., antioxidant and anticancer [36,37]. It was found previously with higher abundance in the algae, e.g., *Himanthalia elongata*, *Laminaria ochroleuca*, *Undaria pinnatifida* [38], *Fucus serratus*, *Fucus vesiculosus* [39,40] or *F. virsoides* [31]. The differences among the fucoxanthin contents of different brown algae species can be associated with different environmental factors and species-inherent characteristics [36,41].

Chlorophyll was not detected in the fractions, but its derivatives devoid of magnesium atoms were found to be more abundant in F4 of both algae (Table 1 and Table 2). A similar finding was reported for *F. virsoides* [31]. The sub-group containing 55 carbon atoms (with the aliphatic side chain) was represented with five compounds (entries 10, 11, 12, 19 and 20, Table 1 and Table 2). In contrast to *Codium adhaerens* [42], the subgroup with 35 carbon atoms (without the aliphatic side chain) was not present. Pheopthyin *b* (entry 19, Table 1 and Table 2) was more abundant in *E. crinita*, particularly in F4 of FdEc, while pheophytin *a* (entry 20, Table 1 and Table 2) was more abundant in F4 of FdEa. Pheophytin *a* has been found previously in notable amounts in different macroalgae [43], like green algae *Enteromorpha prolifera* [36], or *C. adhaerens* [42], as well as brown algae *Sargassum fulvellum* [44] or *F. virsoides* [31]. The other three compounds of this subgroup were pheophytin *a* derivatives characterized by the presence of an additional double bond, carbonyl or hydroxyl group in their composition (entries 10, 11 and 12, Table 1 and Table 2). Pheophytin *a* and related compounds, among other biological activities [45,46], exhibited antioxidant activity for the autooxidation of lipids [47,48].

Terpenes and steroids comprised another group of identified compounds (Table 1 and Table 2). A monoterpene lactone, loliolide (entry 13, Table 1 and Table 2) was found in both algae fractions, being more abundant in F3 of FdEc and FdEa, probably since it is a more polar-containing lactone group and hydroxyl group. It was previously isolated from brown seaweed *Sargassum ringgoldianum*, subsp. *coreanum* [49]. It showed moderate antioxidant activity as well as positive dose-dependent protective effects against H_2_O_2_-induced cell damage [49]. Diterpene isoamijiol (with two free hydroxyl groups, entry 15, Table 1 and Table 2) and its oxidation product C_20_H_30_O_2_ (with one keto group, entry 14, Table 1 and Table 2) were more abundant in F3 of both algae, since they are more polar. Isoamijiol was isolated from the brown seaweed *Dictyota linearis* [50]. Triterpenes β-stigmasterol (entry 16, Table 1 and Table 2) and structurally related sterol (3β,6α)-14-methylergosta-8,24(28)-diene-3,6-diol (entry 17, Table 1 and Table 2) were present in F3 and F4 of both algae. Previously, they were found in *C. adhaerens* [42] and *F. virsoides* [31].

**Figure 3:**
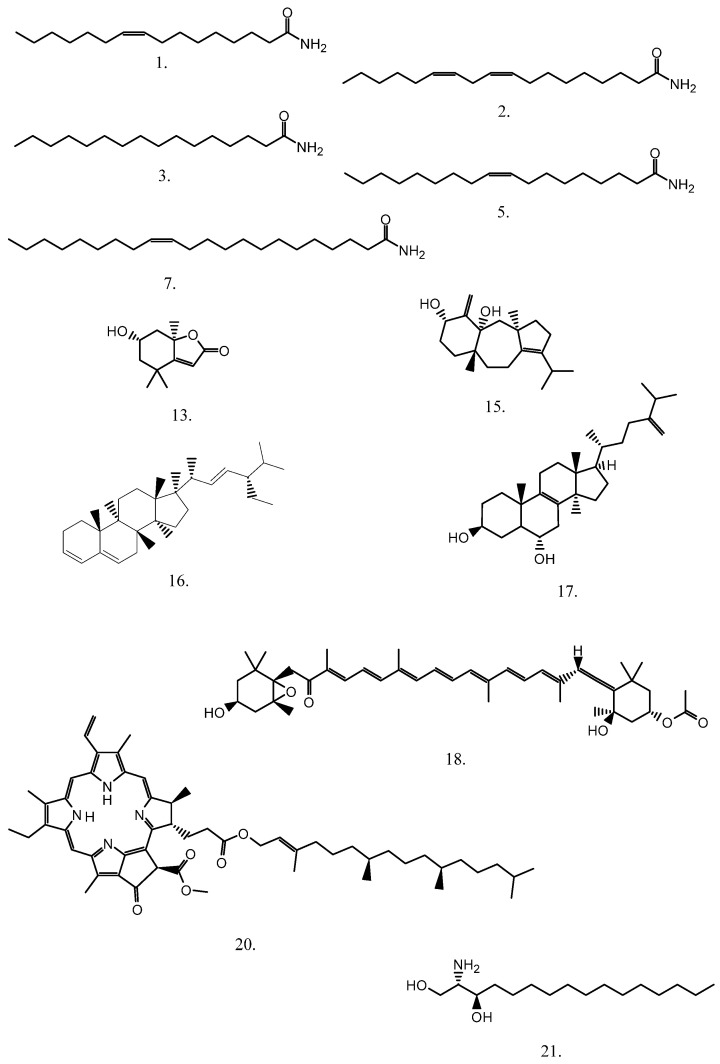
Structural formulas of the most important components identified by HPLC-ESI(+)-HRMS labeled by the numbers depicted in Table 1 and Table 2.

The structural formulas of the most important identified components are presented in Figure 3.

### 2.1. Toxicological Screening of Fractions Obtained from Ericaria Species

#### Zebrafish Embryotoxicity Test

**Figure 4:**
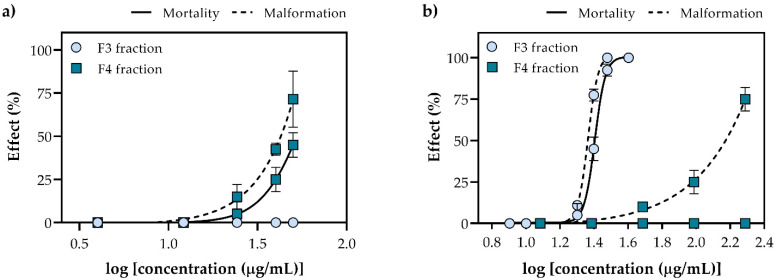
Dose-response curves for the mortality and malformation of zebrafish *Danio rerio* at 96 h of exposure to F3 and F4 fractions of macroalgae (**a**) *E. crinita* (FdEc) and (**b**) *E. amentacea* (FdEa).

The main goal of bioprospecting is the discovery of compound(s) that can be further developed for commercialization, but during the process, toxicological assays and the determination of safety should not be overlooked. Zebrafish has been utilized within this research as one of the most perspective vertebrate models used in different research areas, from genetics, developmental biology, environmental science and (eco)toxicology to drug screening [51]. The results of the conducted embryotoxicity test are presented as concentration-response curves (Figure 4). Among the tested samples, FdEa F3 fraction demonstrated the highest toxicity (50% lethal concentration (LC_50_) = 25.37 ± 1.11 µg/mL, and 50% effective concentration (EC_50_) = 23.12 ± 0.67 µg/mL), followed by FdEc F4 fraction, that revealed a negative effect within a 40–50 µg/mL concentration range, causing 25–45% of mortality and 43–72% of malformations in exposed specimens. Although no mortality was observed during the exposure to FdEa F4 fraction, higher concentrations induced developmental abnormalities, i.e., 75% affected larvae at 194 µg/mL and 25% at 97 µg/mL. The FdEc F3 fraction demonstrated no negative effect on zebrafish survival and development, but one should notice that, in comparison to FdEa F4 fraction, the lower concentration range (4–50 µg/mL) was tested. Morphological abnormalities observed on the tested fractions mainly included pericardial edema and scoliosis. The lethality of the negative control and solvent controls groups was less than 10%.

As can be observed from the results of UHPLC-ESI(+)-HRMS analysis, the tested fractions contained a high amount of fatty acid amides, specifically palmitoleamide, linoleamide, palmitamide, oleamide and erucamide, that might be responsible for the observed toxicity. Bertin et al. [26] demonstrated the presence of seven fatty acid amides (myristamide, palmitamide, oleamide, linoleamide, elaidamide, stearamide and erucamide) in laboratory-grown and field-sampled microalgae *P. parvum* collected during an ichthytoxic harmful algal bloom event, as well as in the culture media and field-collected water. The same authors utilized *Sciaenops ocellatus* and recorded high acute toxicity (100% mortality) upon 6 h of larval exposure to the mixture of seven detected amides (100 ppm) [26]. Such findings indicate that the fatty acid amides detected within this study might be excreted from *Ericaria* species upon the cell lysis, which could translate into elevated mortality and/or a developmental abnormality rate of the exposed zebrafish. Nevertheless, possible synergistic/antagonistic interactions between the detected fatty acid amides, as well as among other molecules in such a complex mixture, should not be excluded. For this reason, further toxicological studies on fatty acid amides, individually and in a mixture, are needed to determine their impact on aquatic vertebrates, and consequently on humans.

### 2.2. Screening of the Antioxidant Activity of Fractions Obtained from Ericaria Species

In the research, the evaluation of the antioxidant activity of two less-polar fractions F3 and F4 of FdEc and FdEa was performed using five different spectroscopic methods: ORAC, FRAP, DPPH, ABTS and Folin–Ciocalteu (F-C) assays.

**Figure 5:**
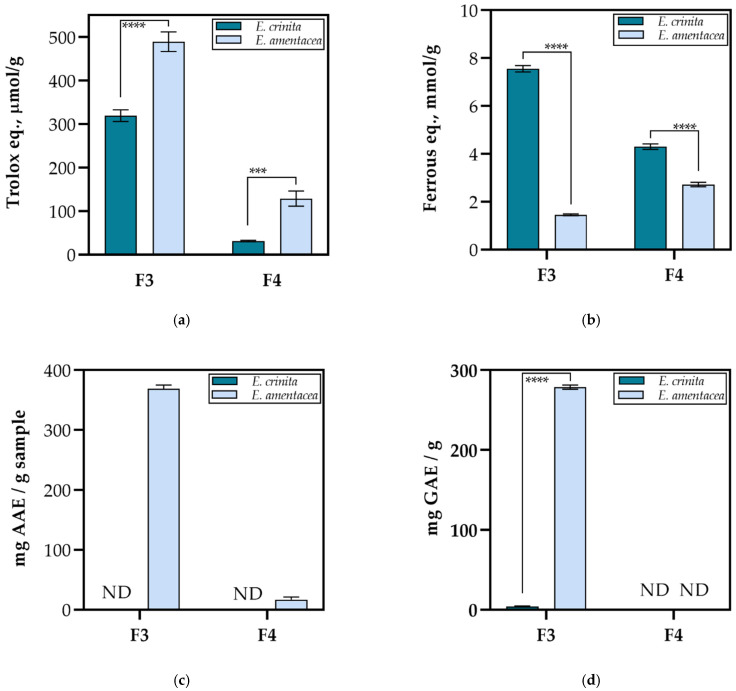
Radical scavenging effect of less-polar fractions from two *Ericaria* macroalgae using (**a**) oxygen radical absorbance capacity (ORAC), (**b**) ferric-reducing antioxidant power (FRAP), (**c**) 2,2-diphenyl-1-picryl-hydrazyl (DPPH) and (**d**) Folin–Ciocalteu in vitro assays (mean ± SD; *n =* 4). An asterisk indicates a significant difference between two *Ericaria* samples for F3 and F4 (*** *p <* 0.001; **** *p <* 0.0001). ND-none determined.

As can be seen from Figure 5, by implementing different methods for the antioxidant activity determination, diverse results were obtained. ORAC assay measures a fluorescent signal from a probe that is quenched in the presence of reactive oxygen species (ROS). The results revealed a 10- and 5-fold higher activity for FdEc and FdEa F3, then F4 fractions, respectively (Figure 5a). Considering the polarity of these fractions, the obtained order could be ascribed to the highest fucoxanthin content in F3 (Table 1 and Table 2). Fucoxanthin is a major carotenoid in seaweeds with already proven antioxidant activity [52]. When compared to our previous research on the endemic brown alga *F. virsoides* [31], it can be concluded that lower values were obtained for both *Ericaria* species.

The FRAP assay was employed because it is based on single electron transfer (SET) and gives a better understanding of the antioxidant reaction mechanism. Interestingly, the results presented in Figure 5b showed an approximately 2-fold (*p* < 0.0001) higher activity for both F3 and F4 fractions of FdEc in comparison to FdEa. However, within the same species, discrepancies can be observed. In FdEc, a higher FRAP value (*p* < 0.0001) was observed for F3 fraction, probably due to the synergistic effect and higher content of found antioxidant compounds (loliolide, isoamijiol and its oxidation product, β-stigmasterol, (3β,6α)-14-methylergosta-8,24(28)-diene-3,6-diol, fucoxanthin, pheophytin *a* and *b*), while in FdEa the opposite result was obtained, i.e., higher activity (*p* < 0.0001) was observed for F4 fraction, indicating that one or more of the found compounds act through the SET mechanism. In this case, it is probably due to (3β,6α)-14-methylergosta-8,24(28)-diene-3,6-diol, the phytosterol abundantly found in F4 of FdEa, since phytosterols have a proven antioxidant activity [53].

The DPPH assay implies a dominant reaction through single electron transfer (SET), but can also react through the transfer of hydrogen atoms (HAT). The inhibition percentage using the DPPH assay for a tested concentration of 1 mg/mL for F3 of FdEa was around 70%, while for F4 this percentage was only 10%. When normalized per gram of the fraction, F3 (369.32 ± 5.85 mg AAE/g fraction) showed a significantly higher (*p* < 0.0001) antioxidant activity than F4 (16.70 ± 4.52 mg AAE/g fraction). For FdEc, no inhibitory percentage could be found using the DPPH assay (Figure 5c).

Further on, although the Folin–Ciocalteu assay is commonly known as a measure of total polyphenolic content, it represents here the rate of the overall antioxidant activity, since the extracts do not contain polyphenols (see Table 1 and Table 2). The activity using the Folin–Ciocalteau assay was only observed for F3 from both species, with a 70-fold higher (*p* < 0.0001) activity for FdEa (278.77 ± 2.67 mg GAE/g) than FdEc (4.12 ± 0.56 mg GAE/g).

**Figure 6:**
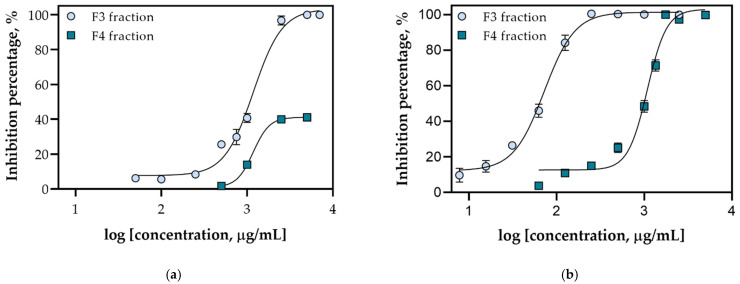
Concentration-inhibition response curves for (**a**) FdEc and (**b**) FdEa F3 and F4 used for the calculation of their antioxidant activity by implementing the ABTS assay.

The fifth method implemented in this research was the reduction of the radical cation by implementing an ABTS assay. Different concentrations of F3 and F4 for both algae were prepared ranging from 0.005 to 5 mg/mL to obtain IC_50_ curves (Figure 6). The IC_50_ values for both samples were calculated as shown in Table 3, with the corresponding confidence interval, slope and coefficient of determination (R^2^). As can be seen, the lowest IC_50_ value, i.e., the highest antioxidant activity was obtained for F3 of FdEa, which is 15 times lower than for F4 of FdEa and F3 of FdEc. This could be explained by the synergistic effect of all found antioxidant compounds with a significantly higher amount of pheophytin *a*. Interestingly, although the highest amount of pheophytin *a* was recorded in F4 of FdEc, its IC_50_ value could not be determined because the upper inhibition plateau could not be reached, i.e., for the highest tested concentrations, the inhibition percentage was around 40%. This discrepancy suggests that, although pheophytin *a* exhibits some antioxidant activity [54], other compounds like fucoxanthin, phytosterols and terpenes have a more significant role in oxidative damage prevention.

The correlation between the antioxidant activity assays for all samples and found antioxidant compounds was preformed using Pearson’s correlation coefficient [55]. The ORAC results showed a higher negative correlation to β-stigmasterol (*r* = −0.770), pheophytin *a* (*r* = −0.857) and pheophytin *b* (*r* = −0.755) and a positive correlation to fucoxanthin (*r* = 0.914). The FRAP results showed a higher positive correlation to loliolide (*r* = 0.719), isoamijiol and its oxidation product (*r* = 0.846 and *r* = 0.879) and (3β,6α)-14-methylergosta-8,24(28)-diene-3,6-diol (*r* = 0.823). The FRAP results showed a higher positive correlation to loliolide (*r* = 0.719), isoamijiol and its oxidation product (*r* = 0.846 and *r* = 0.879) and (3β,6α)-14-methylergosta-8,24(28)-diene-3,6-diol (*r* = 0.823). A moderate negative correlation was found between ABTS results and β-stigmasterol (*r* = −0.532), pheophytin *a* (*r* = −0.415) and pheophytin *b* (*r* = −0.514), while moderate positive correlation was observed with fucoxanthin (*r* = 0.556). However, the significance level was not sufficient enough for any of them and only marginally significant for ORAC results correlated to fucoxanthin (*p* = 0.08), suggesting that the antioxidant activity is the response of a mixture of compounds and their different antioxidant modes of action.

## 3. Materials and Methods

### 3.1. Chemicals

The standards of gallic acid (>97.5%), L-ascorbic acid (≥99%), DPPH (2,2-diphenyl-1-picrylhydrazyl), ABTS (diammonium salt of 2,2′-azino-bis(3-ethylbenzthiazolin-6-yl)sulfonic acid, >99.0%), TPTZ (2,4,6-tripyridyl-S-triazine, ≥98%), dichloro-dihydro-fluorescein diacetate (≥97%, DCF-DA), AAPH (2,2-azobis (2-methylpropionamidine) dihydrochloride, 97%) and 2′,7′-dichlorofluorescin diacetate were purchased from Sigma-Aldrich (St. Louis, MO, USA).

Dimethyl sulfoxide (DMSO, p.a.), methanol (p.a.), ethanol (p.a.), iron (III) chloride (FeCl_3_, p.a.), hydrochloric acid (HCl, p.a.), NaHCO_3_ (p.a.) and Folin–Ciocalteu reagent were obtained from Kemika (Zagreb, Croatia). Hydrogen peroxide (H_2_O_2_, 30%) was purchased from Alkaloid (Skopje, Macedonia) and potassium persulfate (>98%) from Scharlau, (Regensburg, Germany).

The solvents used for SPE were of HPLC grade and were obtained from J.T. Baker (New Jersey, PA, USA).

Acetonitrile with 0.1% (*v*/*v*) formic acid and water with 0.1% (*v*/*v*) formic acid, both hypergrade for HPLC-MS LiChrosolv^®^, were purchased from Supelco Co. (Bellefonte, PA, USA).

### 3.2. Macroalga Samples

*Ericaria crinita* (Duby) Molinari & Guiry 2020 and *Ericaria amentacea* (C.Agardh) Molinari & Guiry 2020 were collected by a single point collection from the Adriatic Sea. *E. crinita* was collected in November 2020 at the southwest coast of Novigrad Sea with the sampling geographical coordinates: 44°12′02″ N; 15°28′51″ E at the depth of 2 m. The sea temperature was 14 °C. *E. amentacea* was collected in April 2021 at the offshore side of Dugi otok, 1 km northwest of Brbišćica Bay, with the sampling geographical coordinates: 43°03′16″ N; 14°59′14″ E. The sea depth was 0.5 m, with the sea temperature at 16 °C. The samples were transferred to the laboratory and washed with water (5 times) and deionized water (2 times), and then the samples were cut in 5–10 mm slices. The slices were frozen in an ultra-low freezer (CoolSafe PRO, Labogene, Denmark) at −60 °C for 24 h. A high vacuum (0.13–0.55 hPa) at −30 °C and 20 °C as the primary and secondary drying temperatures was applied for freeze-drying for 24 h.

### 3.3. Fractionation by Solid-Phase Extraction (SPE)

The freeze-dried *E. crinita* (FdEc) and *E. amentacea* (FdEa) were extracted (10 mL/g solvent:solid ratio) three times with the solvent methanol:dichloromethane (MeOH/DCM, 1:1, *v*/*v*) in an ultrasound-bath (Elma, Elmasonic P 70 H, Singen, Germany; 37 kHz/50 W) for 5 min. The obtained extracts were evaporated by nitrogen (5.0, Messer, Croatia) and were further mixed with C18 powder (40–63 µm, Macherey-Nagel Polygoprep 60–50 C18, Fisher Scientific, MA, USA). The SPE cartridge was conditioned with MeOH and ultrapure water. The obtained dry extracts were then placed on an SPE cartridge (C18, particle size 40 µm, bed weight 1 g, column capacity 6 mL, Agilent Bond Elut, Waldbronn, Germany) and were eluted with different solvents to obtain the fractions F1 to F4 as in our previous paper [11]: F1 (with H_2_O), F2 (with H_2_O/MeOH (1:1, *v*/*v*)), F3 (with MeOH) and F4 (with MeOH/DCM (1:1, *v*/*v*)). Less-polar compounds were obtained in F3 and F4 fractions that were dried by SpeedVac (SPD1030, Thermo Scientific, Waltham, MA, USA) and were stored in the dark at 4 °C.

### 3.4. Ultra-High Performance Liquid Chromatography-High-Resolution Mass Spectrometry (UHPLC-ESI-HRMS) of F3 and F4 Fractions

The UHPLC-HRMS analyses were carried out on an ExionLC AD system (AB Sciex, Concord, ON, Canada) equipped with the ExionLC AD Autosampler, ExionLC AD Pump, ExionLC AD Degasser, ExionLC solvent delivery system, ExionLC AD Column oven and ExionLC Controller combined with a TripleTOF 6600+ (AB Sciex, Concord, ON, Canada) quadrupole-time-of-flight (Q-TOF) mass spectrometer with a Duospray ion source. The chromatographic separation of the compounds in F3 and F4 fractions was achieved using the analytical columns: Acquity UPLC BEH C8 2.1 × 100 mm, particle size 1.7 µm (Waters, Milford, MA, USA) and Acquity UPLC CSH Phenyl-Hexyl, 2.1 × 100 mm, particle size 1.7 µm (Waters, Milford, MA, USA). The mobile phases were water (A) and acetonitrile (B), both containing 0.1% formic acid with the flow rate set at 0.4 mL/min. After the isocratic condition (0.6 min) with 2% of B, the applied gradient elution program was: 0.6–18.5 min (B linear gradient to 100%), 18.5–25 min (100% B). The column temperature was set at 30 °C, and the injection volume was 4 µL.

Mass spectrometry detection was carried out in the positive electrospray ionization (ESI^+^). A collision-induced dissociation (CID) in information-dependent acquisition (IDA) mode of tandem (MS/MS) mass spectra was used for precursor ions with the signal intensities above a 200 cps threshold. The maximum number of precursor ions simultaneously subjected to CID was 15. The ion source parameters were: ESI capillary voltage 5.5 kV and source temperature 300 °C, nebulizing gas (air, gas 1) pressure 40 psi, heater gas (air, gas 2) pressure 15 psi, curtain gas (nitrogen) pressure 30 psi. The recording mass spectra parameters were: 80 V declustering potential, *m*/*z* range 100–1000 (MS) and 20–1000 (MS/MS), and accumulation time 100 ms. Nitrogen was the collision gas, with a collision energy of 40 eV and a spread of 20 eV. The mass scale calibrations (in the MS and MS/MS modes) were done prior to each run with the ESI Positive Calibration Solution 5600 (AB Sciex, Concord, ON, Canada).

The data were processed with ACD/Spectrus Processor 2021.1.0 (ACD/Labs, Toronto, ON, Canada). The compounds’ elemental compositions were determined based on the accurate masses of the corresponding protonated molecules, their isotopic distributions and the product ions *m*/*z* in MS/MS spectra. The identification of detected components was performed based on their elemental compositions, mass spectra and search in the ChemSpider database. The selection among the suggested hits was based on a matching with MS/MS data.

### 3.5. Zebrafish Embryotoxicity Test

Mature zebrafish *Danio rerio* (a wild-type strain obtained from European Zebrafish Resource Center, Karlsruhe Institute of Technology (KIT), Karlsruhe, Germany) were utilized for egg production, following the procedure [32].

A zebrafish embryotoxicity test was conducted in accordance with the OECD Test Guideline, with slight modifications already described in Babić et al. [56]. The embryos (*n* = 20, 4–64 blastomeres) were exposed to a wide range of concentrations (FdEa F3 (8–40 µg/L), FdEa F4 (12–194 µg/L), FdEc F3 and FdEc F4 (4–50 µg/L)). The final solvent concentration (MeOH in F3 fraction and DMSO in F4 fraction) did not exceed 1% [57], which was also the limiting factor during the determination of the concentration range. Artificial water was used as a negative control [58], while MeOH and DMSO (1%) were used as the solvent controls. The exposed zebrafish specimens were incubated at 27.5 ± 0.5 °C (Innova 42 incubator, New Brunswick, Canada). At 96 h of exposure to the tested fractions, mortality and developmental abnormalities were observed using an inverted microscope (Olympus CKX41).

Zebrafish maintenance and spawning were performed in aquaria units approved by the Croatian Ministry of Agriculture and according to the Directive 2010/63/EU. An embryotoxicity test was conducted on the non-protected embryonic/larval stages (up to 96 hpf), which do not require permission by animal welfare commissions [59].

### 3.6. Antioxidant Activity of Tested Fractions

In this study, five methods for the determination of antioxidant activity were employed, including oxygen radical absorbance capacity (ORAC), ferric-reducing antioxidant power (FRAP), a 2,2-diphenyl-1-picryl-hydrazyl (DPPH) assay, the Folin–Ciocalteu method and a reduction of the radical cation (ABTS). The measurements were carried out in triplicates in 96-well plates using a UV/Vis microplate reader (Infinite M200 PRO, TECAN, Switzerland), while all the results were expressed as mean ± standard deviation (*n* = 4).

The reduction of the radical cation assay (ABTS) was measured by spectroscopy at 734 nm [60] with slight modifications. The ABTS radical cation stock solution was prepared by mixing 7 mM ABTS and 2.45 mM potassium persulfate in the same ratio. The obtained mixture was allowed to react at room temperature in the dark for 17 h. The working solution of the ABTS radical cation was adjusted to an absorbance of 0.700 ± 0.02. The reaction mixture was prepared by adding the sample and an ABTS working solution, achieving the inhibition percentage between 10% and 100% (the blank was represented by the used solvent for each fraction). The results were expressed as milligram Trolox equivalents per gram of sample (mg TE/g). Moreover, an IC_50_ curve was constructed for both samples.

The oxygen radical absorbance capacity (ORAC) was determined as described by Huang and colleagues [61], with some modifications. A 25 μL diluted sample was added in black 96-well flat-bottom plates. Afterwards, 150 μL of a DCF-DA solution (1:1000, *v*/*v* in 25 mL 75 mM PBS) was added, followed by incubation at 37 °C for 30 min in the shaking incubator (New Brunswick, Innova 42). To start the reaction, 25 μL AAPH was added to the mixture, and the loss of fluorescence was monitored every 5 min for 180 min. The excitation wavelength was 485 nm and the emission wavelength was 528 nm, with an optimal fluorescence gain of 209. The results were expressed as μmol Trolox equivalents (TE)/g of the fraction.

For the FRAP assay [62], the FRAP regent must first be prepared by mixing the equal volumes of a 10 mM TPTZ solution in 40 mM HCl and an aqueous 20 mM FeCl_3_ solution. Then, the mixture was diluted five times in 0.25 M acetate buffer (pH 3.6) and heated to 37 °C. The 100 μL of the sample was mixed with 3.9 mL of the FRAP reagent, and the absorbance was determined at 593 nm after the incubation at 37 °C for 10 min. The results are expressed as mmol/g ferrous equivalents.

For the DPPH radical scavenging assay [63], the volume of 25 μL of the prepared sample was mixed with 200 μL of methanol and the prepared DPPH reagent in methanol (240 μg/mL). The reaction mixture was kept in the dark for 30 min, after which the absorbance of the solution was measured at 490 nm. The results were expressed as milligram ascorbic acid equivalents per gram of the sample (mg/gAAE).

The Folin–Ciocalteu method was used [64] with some adaptations. Briefly, 100 μL of the sample was mixed with 750 μL of a 10-fold diluted Folin–Ciocalteu reagent. After 5 min of incubation at room temperature, 750 μL of sodium bicarbonate solution (60 g/L) was added to the mixture and incubated in the dark at room temperature for 90 min. Absorbance was measured at 750 nm, and the results were expressed as mg gallic acid equivalent (GAE) per g of the sample.

### 3.7. Statistical Analysis

GraphPad Prism software version 8 was used for statistical analysis and graph presentation. *p* ≤ 0.05 was used as a cut-off value of statistical significance throughout the manuscript. Prior to LC_50_/EC_50_ determination, the obtained values were subjected to logarithmic transformation. A one-way analysis of variance (ANOVA) was performed to examine the significance between tested samples, as well as among treatments. The correlation between the antioxidant assay results and the chemical composition was tested through Pearson correlation analysis.

## 4. Conclusions

*E. crinita* and *E. amentacea* from the Adriatic Sea (Croatia) were investigated for the first time with respect to the presence of less-polar compounds. After fractionation by solid-phase extraction (SPE), the major identified compounds by UHPLC-ESI-HRMS were: amides of higher aliphatic acids (palmitoleamide, linoleamide, oleamide and erucamide) and related compounds, carotenoid (fucoxanthin), chlorophyll derivatives (pheophytin *a* and *b* and their derivatives) and higher terpenes (loliolide, isoamijiol and its oxidation products), β-stigmasterol and (3β,6α)-14-methylergosta-8,24(28)-diene-3,6-diol). Fucoxanthin was found to be the most abundant in F3 of FdEc and FdEa. Pheopthyin *b* was more abundant in F4 of FdEc, while pheophytin *a* was more abundant in F4 of FdEa. Loliolide and isoamijiol were found in both algae fractions, being more abundant in F3.

The results of toxicological testing obtained within this study emphasize the need to employ model organisms for determining the toxicological effect of bioactive molecules, all in order to provide answers on their safety for non-target organisms and, consequently, humans.

Higher antioxidant activities of FdEa in comparison to FdEc fractions were found by implementing all five assays (ABTS, ORAC, FRAP DPPH, Folin–Ciocalteu), with inhibitory concentrations of 0.072 and 1.177 mg/mL for the fractions, respectively. By correlating the results of the antioxidant analysis with the chemical composition, it was determined that the synergistic effect of different compounds, as well as their modes of action, impact the antioxidant response. The high antioxidant activity of *E. amentacea* suggests its potential as a source of natural antioxidants. The correlations between two Ericaria species show that the same species has a different composition and consequently diverse activities.

## Figures and Tables

**Table 1:** Major identified compounds in F3 and F4 fractions of *E. crinita* and their tentative identification by UHPLC-ESI(+)-HRMS.

No.	Compound	Elemental Composition	[M + H]^+^	Error ** (ppm)	A			B		
					RT (min)	Area (Counts)		RT (min)	Area (Counts)	
						F3	F4		F3	F4
1.	Palmitoleamide	C_16_H_31_NO	254.24784	0.5	13.609	1108116	320801	13.085	1309202	3128998
2.	Linoleamide	C_18_H_33_NO	280.26349	0.7	14.086	1390454	3166360	13.546	1574298	3629020
3.	Palmitamide	C_16_H_33_NO	256.26349	0.9	14.460	5498614	3834048	13.818	6396427	3991527
4.	2,3-Dihydroxypropyl palmitate	C_19_H_38_O_4_	331.28429	4.0	14.817	238983	16797	14.129	295785	21659
5.	Oleamide	C_18_H_35_NO	282.27914	0.5	14.868	25284826	54228974	14.209	26096208	53855452
6.	2,3-Dihydroxypropyl stearate	C_21_H_42_O_4_	359.31559	4.0	15.974	330917	58977	15.162	409784	70222
7.	Erucamide	C_22_H_43_NO	338.34174	0.1	16.981	5433654	1438264	16.084	4682127	1767616
8.	2-Hydroxypropyl stearate	C_21_H_42_O_3_	343.32067	1.5	17.269	38981	1214	16.254	46373	1025
9.	(2*S*)-1-Hydroxy-3-(tetradecanoyloxy)-2-propanyl (9*Z*)-9-octadecenoate	C_35_H_66_O_5_	567.49830	1.3	19.231	285866	10547	17.976	348859	14426
10.	3-Phorbinepropanoic acid, 3,4-didehydro-9-ethenyl-14-ethyl-24,25-dihydro-21-(methoxycarbonyl)-4,8,13,18-tetramethyl-20-oxo-, (2*E*)-3,7,11,15-tetramethyl-2-hexadecen-1-yl ester	C_55_H_72_N_4_O_5_	869.55755	1.1	20.373	4307	1110897	19.066	5350	1005380
11.	Methyl (3*R*,10*Z*,14*Z*,20*Z*,22*S*,23*S*)-12-ethyl-3-hydroxy-13,18,22,27-tetramethyl-5-oxo-23-(3-oxo-3-{[(2*E*,7*R*,11*R*)-3,7,11,15-tetramethyl-2-hexadecen-1-yl]oxy}propyl)-17-vinyl-4-oxa-8,24,25,26-tetraazahexacyclo [19.2.1.1^6,9^.1^11,14^.1^16,19^.0^2,7^]heptacosa-1(24),2(7),6(27),8,10,12,14,16,18,20-decaene-3-carboxylate	C_55_H_74_N_4_O_7_	903.56303	1.8	20.441	45606	2940710	19.987	38274	2268701
12.	3-Phorbinepropanoic acid, 9-acetyl-14-ethylidene-13,14-dihydro-21-(methoxycarbonyl)-4,8,13,18-tetramethyl-20-oxo-, 3,7,11,15-tetramethyl-2-hexadecen-1-yl ester	C_55_H_74_N_4_O_6_	887.56811	1.9	20.594	332532	11277103	19.953	401031	13903240
13.	Loliolide	C_11_H_16_O_3_	197.11722	1.2	6.225	88150	4603	6.323	86765	5693
14.	Isoamijiol oxidation product *	C_20_H_30_O_2_	303.23186	6.2	14.544	378362	2372	14.328	352017	2346
15.	(3a*R*,4a*R*,6*S*,8a*R*)-1-Isopropyl-3a,8a-dimethyl-5-methylene-2,3a,4,5,6,7,8,8a,9,10-decahydrobenzo[f]azulene-4a,6(3H)-diol (Isoamijiol)	C_20_H_32_O_2_	305.24751	0.5	15.974	122683	17514	15.522	104432	22138
16.	β-Stigmasterol	C_29_H_46_	395.36723	1.6	18.276	36308	364233	17.634	33268	459395
17.	(3β,6α)-14-Methylergosta-8,24(28)-diene-3,6-diol	C_29_H_48_O_2_	429.37271	3.4	18.395	784665	96119	17.480	980078	117567
18.	Fucoxanthin	C_42_H_58_O_6_	659.43062	2.1	14.873	2232648	3500	15.122	2697740	4095
19.	Pheophytin *b*	C_55_H_72_N_4_O_6_	885.55246	8.7	20.134	39490	467361	19.765	40024	391298
20.	Pheophytin *a*	C_55_H_74_N_4_O_5_	871.57320	2.1	20.815	5854	61538472	20.106	7418	57737924
21.	Hexadecasphinganine	C_16_H_35_NO_2_	274.27410	1.0	9.507	3186185	2813931	7.789	3952304	3361364

A-USP L7 (Acquity BEH C8) column.; B-USP L11 (Acquity CSH phenyl-hexyl) column; *—exact compound not determined; **—the smallest error for both columns.

**Table 2:** Major identified compounds in F3 and F4 fractions of *E. amentacea* and their tentative identification by UHPLC-ESI(+)-HRMS.

No.	Compound	Elemental Composition	[M + H]^+^	Error ** (ppm)	A			B		
					RT (min)	Area (Counts)		RT (min)	Area (Counts)	
						F3	F4		F3	F4
1.	Palmitoleamide	C_16_H_31_NO	254.24784	1.0	13.628	592811	2300496	13.05	497757	1965778
2.	Linoleamide	C_18_H_33_NO	280.26349	2.0	14.088	479266	2697537	13.509	575290	2671906
3.	Palmitamide	C_16_H_33_NO	256.26349	1.5	14.48	706476	3340508	13.781	628067	2844809
4.	2,3-Dihydroxypropyl palmitate	C_19_H_38_O_4_	331.28429	3.1	14.82	18264	50976	14.105	17392	63085
5.	Oleamide	C_18_H_35_NO	282.27914	1.5	14.888	4871406	36851920	14.189	6088715	30534284
6.	2,3-Dihydroxypropyl stearate	C_21_H_42_O_4_	359.31559	1.5	15.994	21985	83888	15.14	19816	86090
7.	Erucamide	C_22_H_43_NO	338.34174	0.6	16.994	2971406	1697387	16.057	2470961	1417294
8.	2-Hydroxypropyl stearate	C_21_H_42_O_3_	343.32067	0.0	17.181	896	7775	16.327	1111	9880
9.	(2*S*)-1-Hydroxy-3-(tetradecanoyloxy)-2-propanyl (9Z)-9-octadecenoate	C_35_H_66_O_5_	567.49830	1.9	19.225	185815	176355	17.948	226104	218486
10.	3-Phorbinepropanoic acid, 3,4-didehydro-9-ethenyl-14-ethyl-24,25-dihydro-21-(methoxycarbonyl)-4,8,13,18-tetramethyl-20-oxo-, (2*E*)-3,7,11,15-tetramethyl-2-hexadecen-1-yl ester	C_55_H_72_N_4_O_5_	869.55755	0.0	20.37	1042	1968855	20.01	866	1678772
11.	Methyl (3*R*,10*Z*,14*Z*,20*Z*,22*S*,23*S*)-12-ethyl-3-hydroxy-13,18,22,27-tetramethyl-5-oxo-23-(3-oxo-3-{[(2*E*,7*R*,11*R*)-3,7,11,15-tetramethyl-2-hexadecen-1-yl]oxy}propyl)-17-vinyl-4-oxa-8,24,25,26-tetraazahexacyclo [19.2.1.1^6,9^.1^11,14^.1^16,19^.0^2,7^]heptacosa-1(24),2(7),6(27),8,10,12,14,16,18,20-decaene-3-carboxylate	C_55_H_74_N_4_O_7_	903.56303	3.3	20.437	48785	1044658	19.981	40622	876040
12.	3-Phorbinepropanoic acid, 9-acetyl-14-ethylidene-13,14-dihydro-21-(methoxycarbonyl)-4,8,13,18-tetramethyl-20-oxo-, 3,7,11,15-tetramethyl-2-hexadecen-1-yl ester	C_55_H_74_N_4_O_6_	887.56811	1.3	20.591	45728	1294640	19.964	37547	1053061
13.	Loliolide	C_11_H_16_O_3_	197.11722	5.6	6.222	32631	14304	6.403	27206	13071
14.	Isoamijiol oxidation product *	C_20_H_30_O_2_	303.23186	0.4	14.534	48292	8398	14.427	51830	10486
15.	(3a*R*,4a*R*,6*S*,8a*R*)-1-Isopropyl-3a,8a-dimethyl-5-methylene-2,3a,4,5,6,7,8,8a,9,10-decahydrobenzo[f]azulene-4a,6(3H)-diol (Isoamijiol)	C_20_H_32_O_2_	305.24751	5.3	15.959	22350	12061	15.58	27465	14464
16.	β-Stigmasterol	C_29_H_46_	395.36723	1.9	18.288	19126	670276	17.607	16495	562142
17.	(3β,6α)-14-Methylergosta-8,24(28)-diene-3,6-diol	C_29_H_48_O_2_	429.37271	2.2	18.39	171540	273113	17.47	143026	279999
18.	Fucoxanthin	C_42_H_58_O_6_	659.43062	0.4	15.125	2138127	89864	14.851	1811467	86705
19.	Pheophytin *b*	C_55_H_72_N_4_O_6_	885.55246	0.2	20.148	20692	155992	19.776	21058	131321
20.	Pheophytin *a*	C_55_H_74_N_4_O_5_	871.57320	0.9	20.829	240373	41982766	20.116	199581	46894376
21.	Hexadecasphinganine	C_16_H_35_NO_2_	274.27410	0.4	9.473	1226868	3731490	7.762	1053691	3149871

A–USP L7 (Acquity BEH C8) column.; B–USP L11 (Acquity CSH phenyl-hexyl) column; *—exact compound not determined; **—the smallest error for both columns.

**Table 3:** Dose-inhibition results using the ABTS in vitro assay (*n* = 4) to obtain the half-maximal inhibitory concentration (IC_50_) with the presented confidence intervals, Hillslope and R^2^ value.

Sample	IC_50_ Value, mg/mL	Confidence Interval (95%)	Hillslope
FdEc F3	1.177	1.049–1.346	2.58
FdEc F4	ND *	-	-
FdEa F3	0.072	0.067–0.077	2.477
FdEa F4	1.060	0.986–1134	3.944

* ND—none defined.

## Data Availability

Data are contained within the article.
